# Vanadium Methyl-Bipyridine Organoligand and its Influence on Energy Balance and Organs Mass

**DOI:** 10.1007/s12011-014-0064-y

**Published:** 2014-07-12

**Authors:** Mirosław Krośniak, Renata Francik, Agnieszka Wojtanowska-Krośniak, Cinzia Tedeschi, Małgorzata Krasoń-Nowak, Joanna Chłopicka, Ryszard Gryboś

**Affiliations:** 1 Department of Food Chemistry and Nutrition, Jagiellonian University Medical College, 9 Medyczna Str, 30-688 Krakow, Poland; 2 Department of Bioorganic Chemistry, Jagiellonian University Medical College, 9 Medyczna Str, 30-688 Krakow, Poland; 3 State Higher Vocational School, Institute of Health, Staszica 1 Str, 33-300 Nowy Sącz, Poland; 4 Department of Food Chemistry and Nutrition, Student at the Faculty of Pharmacy Nutritional and Health Sciences–Calabria University, Arcavacata di Rende, Italy; participant of Erasmus Program in the, Jagiellonian University, Medical College, Krakow, Poland; 5 Faculty of Chemistry, Jagiellonian University, 3 Ingardena Str, 30-060 Krakow, Poland

**Keywords:** Vanadium, High-fructose diet, High-fat diet, Rat, Body mass growth

## Abstract

In the treatment of lifestyle diseases, including metabolic syndrome and type 2 diabetes, it is important to lower body mass and fat tissue, and consequently, to increase insulin-sensitivity. Unfortunately, it often happens that low-energy diet which would lower overweight is not observed and, thus, it does not bring the expected effects. This paper discusses the influence of three diets—control, high-fructose, and high-fatty diet—on absorption of energy from food in order to transform it into body mass. The kJ/g ratio which describes this process has been calculated. In the tested diets, the addition of fructose (79.13 ± 2.47 kJ/g) or fat (82.48 ± 2.28 kJ/g) results in higher transformation of energy into body mass than in the case of control diet (89.60 ± 1.86 kJ/g). The addition of Na[VO(O_2_)_2_(4,4′-Me_2_-2,2′-bpy)]•8H_2_O (where 4,4′-Me_2_-2,2′-bpy = 4,4′-dimethyl-2,2′-bipyridine) results in statistical increase of that ratio: fructose diet (86.88 ± 0.44 kJ/g), fat diet (104.68 ± 3.01 kJ/g), and control diet (115.98 ± 0.56 kJ/g), respectively. Fat diet statistically influences the decrease of kidney mass in comparison to the other diets. The application of the tested vanadium compound results also in the statistical decrease of the fatty liver caused by fructose and fat diet.

## Introduction

Diabetes, cardiovascular disease, obesity, and depression are among the most frequently diagnosed civilization diseases [1–3]. The root of these diseases is improper diet and metabolic changes in the organism, associated with low physical activity. It is mainly the consumption of high monosaccharides, simple sugars, and high saturated fat acids combined with low physical activity that can provoke the abovementioned illnesses [4–6]. High processed food is likely to be important in this process. For this reason, currently, many dietary methods (diets) are used in prevention of these risks [7–9]. Frequently, nutritional regime is difficult to maintain for a long time, and drugs and surgical help are necessary [10, 11]. There are known many legal and illegal organic compounds which can increase metabolism [12–14]. Similar results have also been observed for inorganic compounds, e.g., chromium [15, 16]. One of the new possibilities is treatment of all these diseases with vanadium compounds. Vanadium has been investigated as a potential microelement useful in diabetes treatment for more than 30 years [17–20]. Undesirable effects such as nausea, diarrhea, stool discoloration, abdominal cramps, etc. are sometimes observed during the treatment with vanadium compounds [21]. As the administered doses of vanadium compounds are usually high, they can give toxic effects. The differences observed between positive and toxic effect are very important in determining the therapeutic doses of the tested vanadium compounds [22–26]. At present, in the research of vanadium compounds, the minimal therapeutic doses are used to minimize the toxic or side effects. For this moment, bis(maltolato)oxovanadium(IV) (BMOV) is frequently used as a reference substance because the number of experiments with this chemical substance is the highest [27–29]. Other compounds are not investigated in all aspects as BMOV but sometimes have better properties in selected aspects, especially small toxicity with the same therapeutic effect [6, 7]. Vanadium coordination compounds with organic ligands showed lower toxic effect in comparison to simple inorganic compounds (e.g., NaVO_3_ and VOSO_4_) and, therefore, researches concentrate now on different organoligand vanadium compounds [30]. Although the body mass growth during vanadium treatment was smaller than in control vanadium not treated animals, the authors of this paper are not aware of any works concerning the influence of the tested vanadium compounds on complete energy balance. Many researchers believe that small body growth during the experiment was associated with potential toxic effect or problems of gastrointestinal tract. Our observations and energy balance can show a potential new action of vanadium in metabolism regulation.

## Materials and Methods

### Vanadium Complex

**Fig. 1:**
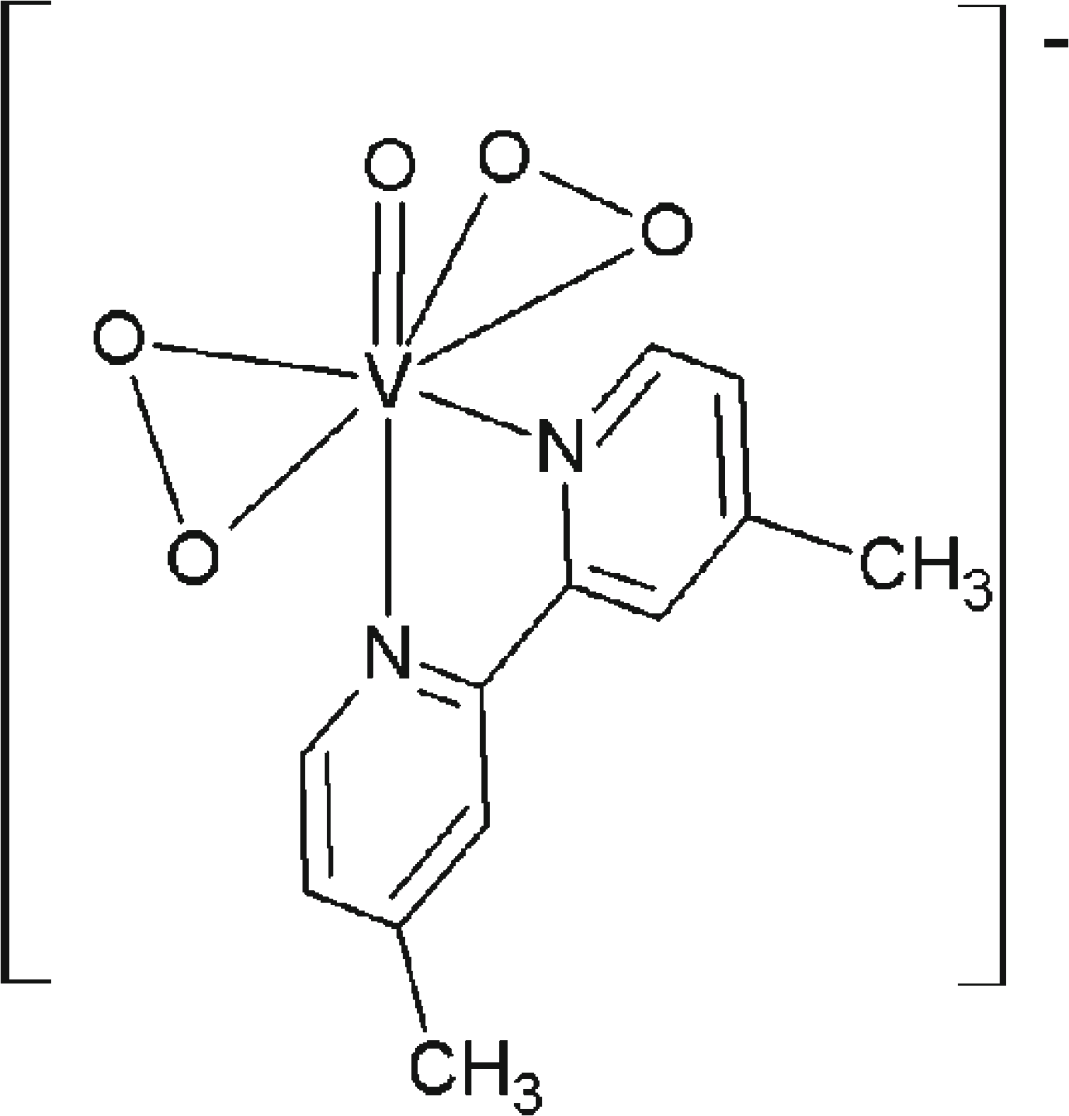
Chemical structure of 4,4′-Me_2_-2,2′-bpy = 4,4′-dimethyl-2,2′-bipyridine (Na[VO(O_2_)_2_(4,4′-Me_2_-2,2′-bpy)]•8H_2_O) used in present investigation

Bisperoxo complex of vanadium (V)–Na[VO(O_2_)_2_(4,4′-Me_2_-2,2′-bpy)]•8H_2_O (MW = 481.94 g/mol). The synthesis was carried out by modified method described in literature [31]. Ten millimoles of NaVO_3_ was dissolved in molar excess of 10 % H_2_O_2_ (molar ratio of H_2_O_2_ to vanadium is equal 1:3). To the obtained clear yellow solution, cooled in the ice bath, 20 ml of ethanolic solution containing 10 mmol of proper 4,4′-Me_2_-2,2′-bpy (4,4′-dimethyl-2,2′-bipyridine) was added dropwise with constant stirring. Temperature of the reaction mixture did not exceed 10 °C during the synthesis. Afterwards, 50 ml of cooled ethanol was added to precipitate yellow crystals. The solid phase was filtered off via a glass frit and washed with 10 ml of cold ethanol. Obtained vanadium complex was dried in the air, in a dark place, for 24 h, then it was collected and stored in the refrigerator. Purity of obtained complex was confirmed by microanalysis, spectroscopic methods: ^1^H NMR and IR. The structure of complex anion is presented in Fig. 1.

### Food for Animals

Animals used in the experiment had unlimited access to food and drinking water. Three different diets were used in the experiment: normal, high-fructose, and high-fatty. All of these diets were prepared especially for this experiment in the Department of Food Chemistry and Nutrition Jagiellonian University Medical College. Table 1 below presents the used components.

**Table 1:** Percentage composition of the diets used in the experiment

Component	Normal diet (C) (%)	High-fructose diet (Fr) (%)	High-fatty diet (Ft) (%)
Starch	62.0	31.0	32.0
Casein	20.0	20.0	20.0
Rapeseed oil	5.0	5.0	5.0
Fructose	0.0	31.0	0.0
Lard	0.0	0.0	30.0
Calcium carbonate	2.8	2.8	2.8
Ca_3_(PO_4_)_2_	2.9	2.9	2.9
Lecithin	1.0	1.0	1.0
NaCl	0.3	0.3	0.3
Cellulose	4.7	4.7	4.7
Minerals and vitamins mix.	1.0	1.0	1.0
MgO	0.07	0.07	0.07
K_2_SO_4_	0.23	0.23	0.23
Caloric kJ/g	16.024	16.024	22.555

### Animals

The experiment was conducted on 3-month-old male Wistar rats, weighing 250 ± 15 g and caged in the temperature of 23 °C, humidity 50–60 %, and light dark cycle (12/12 h). Each group consisted of six animals. During 5 weeks of the experiment, each group of animals was fed a different diet: control (C), fructose (Fr), fatty (Fa), control with vanadium (CV), fructose with vanadium (FrV), and fat with vanadium (FaV). The diet composition is shown in Tab. 1. The animals had unlimited access to food and water. The groups of the animals with vanadium were treated with vanadium complex (Na[VO(O_2_)_2_(4,4′-Me_2_-2,2′-bpy)]•8H_2_O) in the dose of 20 mg/kg one time daily (at 9 a.m.) during 5 weeks directly to stomach by gavage. Food and water consumption were measured three times per week (Monday, Wednesday, and Friday) during 5 weeks of the experiment. Body mass was also recorded at the same time. After 5 weeks of the experiment, the animals were anesthetized and organs—brain, heart, liver, testicles, kidneys, lung, spleen, and pancreas—were isolated and immediately weighed with an accuracy to 1 mg on the Sartorius basic scales.

### Statistics

All statistical analysis was made using Statistica 10 software and nonparametric test with *p* < 0.05 as statistical significance. In Table 2 are presented results as median ± standard deviation.

**Table 2:** Results for measured parameters in different group of animals (median ± standard deviation)

Group	Ratio energy /g of body growth KJ/g	Body mass growth (g)	Kidney percentage (%)	Liver percentage (%)	Spleen percentage (%)	Heart percentage (%)	Liver steatosis (%)
C	89.60 ± 1.86	110.5 ± 15.4	0.647 ± 0.052	3.892 ± 0.424	0.188 ± 0.035	0.296 ± 0.015	20.02 ± 5.58
Fr	79.13 ± 2.47	132.0 ± 12.0	0.688 ± 0.054	4.003 ± 0.288	0.190 ± 0.022	0.281 ± 0.009	28.75 ± 7.04
Fa	82.48 ± 2.28	125.0 ± 21.3	0.557 ± 0.027	3.491 ± 0.171	0.176 ± 0.006	0.268 ± 0.016	27.00 ± 5.48
CV	115.98 ± 0.56	84.5 ± 13.3	0.645 ± 0.049	3.786 ± 0.278	0.175 ± 0.022	0.278 ± 0.011	21.06 ± 5.94
FrV	86.88 ± 0.44	113.0 ± 19.4	0.731 ± 0.036	3.850 ± 0.241	0.177 ± 0.008	0.270 ± 0.005	20.59 ± 1.41
FaV	104.68 ± 3.01	95.0 ± 28.2	0.623 ± 0.050	3.655 ± 0.110	0.161 ± 0.016	0.270 ± 0.012	18.67 ± 2.39

## Results

(A) It was observed during the experiment that the animals in the tested groups consumed similar quantities of food. However, the body mass increase in various groups was different. A decision was made to calculate the kJ/g ratio of body mass increase, which would allow determining how the tested diets and vanadium influenced the body mass growth in the tested animals. The influence was assessed based on the quantity of kilojoules delivered in the food consumed by all animals in a given group, divided by the aggregate body mass increase of the animals in that group (Table 2). In the case of vanadium nontreated animals, the lowest value of the kJ/g ratio was observed in the fructose and fatty diets (Fr and Fa). The body weight gain of about 1 g in the animals from the control group (C) required statistically more kJ in comparison to the fructose and fatty diet. Vanadium treatment in all the used diets caused statistically significant increase of the kJ/g ratio in comparison to vanadium not treated animals. The smallest differences were observed between Fr and FrV groups. The biggest differences were observed between C versus CV and Fa versus FaV groups.

(B) Body mass growth (Table 2) was calculated as a difference in weight of the animal at the end and at the beginning of the experiment. Fructose (Fr) and fatty diet (Fa) increased body growth in comparison to the control animals (C) in all vanadium not treated animals but the result was not statistically significant. In the case of vanadium-treated animals, significant difference was observed for the normal (CV) and the fructose diet (FrV). The fatty diet (Fa) also increased body growth in comparison with the normal diet (CV), but not statistically. Vanadium treatment slows the growth rates of body weight compared to the animals without vanadium treatment.

(C) Kidney percentage in all body mass: Comparing the obtained results (Table 2), a clear influence of diet on the kidney mass percentage share in the total animal body mass can be observed. Fatty diet (Fa) decreased the kidney mass percentage in comparison to the control group but the result was not statistically significant. In the Fr group, a statistically significant increase of the kidney mass percentage was observed in comparison to the fatty diet group. No influence of the tested vanadium compound on the tested parameter was observed.

(D) Liver percentage in all body mass: The diet influence on the analyzed organ was observed also in the case of the liver percentage share in the total animal body mass (Table 2). Fatty diet (Fa) decreased—although not statistically—the liver mass in comparison to the control group (C) and statistically in comparison to the fructose diet group. In the vanadium-treated animal groups, practically no differences were observed. Additionally, vanadium influence was observed in fatty diet. In the vanadium compound treated animals with fatty diet, statistical increase of the liver mass percentage was observed.

(E) Spleen percentage in all body mass: The spleen mass percentage in proportion to the body mass was the lowest in the FaV group and the highest in the Fr group (Table 2). However, no statistically significant differences were observed for that parameter. One can only point to a tendency of the indicator to decrease in case of fatty diet. Adding the tested vanadium compound to fatty diet intensifies the indicator decrease.

(F) Heart percentage in all body mass: The heart mass percentage share in the total body mass was the highest for the control group animals (C) and the lowest for the fatty diet animal group (Table 2). A tendency of that indicator to decrease was observed in all animal groups receiving the tested vanadium compound. The differences were close to being statistically significant for the C group in comparison to CV and Fr in comparison to FrV.

(G) Liver steatosis in different groups of animals: In the Fa animal group, that parameter was also insignificantly lower (Table 2). A clear influence of the Fr and Fa diets on liver steatosis in comparison to the control group (C) was observed (verging on being statistically significant). Adding vanadium to the fructose and fatty diet resulted in statistically significant decrease of the liver steatosis.

## Discussion

In the past, one of the signs of high social status was a rounded posture. This meant that the owner did not lack food and could protect his or her family. During the centuries, people had many problems with the provision of adequate calories [32]. Crop failure was often the cause of famine. In addition, average physical activity was significantly greater than at present. The technological development of modern agriculture has increased food surpluses [33]. The use of machines in the course of industrial production reduced demand for physical work. Both these factors: the availability of food and low physical activity, are a major cause of obesity and diseases associated with this problem [34]. In some countries, e.g., USA or Mexico, the percentage of obese population (BMI > 30 kg/m^2^) is higher than 20 % [35]. Obesity increases the risk of hypertension, diabetes, metabolic disorders associated with distorted lipid metabolism, and fat redistribution (abdominal type obesity is very unfavorable) [36]. In the world, the awareness of this risk increases and various programs are developed to combat this threat. Deficit of energy associated with the reduction of calorie intake plays a principal role in lowering body mass but compliance with dietary recommendations may not be possible for a lot of people for different reasons. One of the possibilities is supplementation of products which increase body metabolism [10, 37]. It is of great importance for persons more than 40 years old whose metabolism is slower that in young people as in this period of life problems with glucose tolerance begin. For the moment, some products are used: thermogenics such as l-carnitine [38], chromium picolinate [15, 16], chitosan [39], or chlorogenic acid-ACG [40], hydroxycitric acid-HCA [41], gymnemic acid [42], which belong to amylase inhibitors which reduce the absorption of glucose and block the formation of fat from carbohydrates. With respect to these products, metals such as chromium or vanadium showed interesting effects. Vanadium and its compounds have been tested since 1980s as a potential antidiabetic substance which can help in diabetes treatment [17–21]. During experiments with this element, a decrease of body mass grow was observed in comparison to the vanadium not treated animals. Many researches thought that this effect was associated with toxicity of vanadium because they did not make energetic calculation between consumed energy and body mass growth. In our study, three different diets were tested: control, one with 30 % of fructose and one with 30 % of saturated animal fat (lard). During 5 weeks of the experiment, clear effect of the used diets C, Fa, Fr, CV, FaV, and FrV on the calculated ratio was observed: the number of absorbed calories to weight gain (kJ/g). Addition of fructose or lard has an influence on the metabolism and transformation of energy from animal feed to body mass. The ratio calculated for fructose was 79.13 ± 2.47 kJ/g and it was similar to the ratio for lard addition which was 82.48 ± 2.28 kJ/g. For the control feed, the ratio was 89.60 ± 1.86 kJ/g and the result was statistically significant in comparison to both diets with fructose or fat. More interesting observations were reported on vanadium treatment in these three diets where the calculated ratio statistically increased. Vanadium evidently influenced metabolism and the transformation of energy into body mass of the investigated animals. In control diet, the vanadium treatment increased the ratio from 89.60 ± 1.86 to 115.98 ± 0.56 kJ/g. For fatty diet, the ratio increased from 82.48 ± 2.28 to 104.68 ± 3.01 kJ/g. The smallest—but significant—increase was observed for the calculated ratio for fructose diet where vanadium increased this coefficient from 79.13 ± 2.47 to 86.88 ± 0.44 kJ/g. The mechanism of metabolism action was not studied in this experiment but our observations open possibilities for future investigations associated with the observed effect. For better understanding of the possibilities into what the energy from food was transformed (for example: increase of protein production, body temperature, physical activation, or other ways), it is necessary to prepare the next experiment which can provide answers to these hypotheses. Comparing the obtained results from our experiment with works of other authors is also difficult because they did not make this type of calculation. Based on the results of the work of Chung et al. [43] and Shi et al. [44], after the ratio (kJ/g) calculation, our results are similar to their observations. High-fat diet treatment increases energy processing into body weight. Vanadium treatment not only influences body mass and transformation of energy from animal feed into body mass. Also the size of some organs is associated with diets and vanadium. The biggest differences are reported for the kidney and the liver, smaller for the spleen and the heart. The percentage of kidney weight in the whole body was the lowest in both fatty diet groups: with vanadium treated and not treated animals. In both fructose groups, the percentage of kidney weight in whole body mass was the highest.

In the case of the liver, differences between groups in vanadium not treated animals are observed. The percentage of liver weight in the whole body mass was the smallest in the fatty diet group and it was statistically significant in comparison to the fructose group. In the vanadium-treated groups, differences between diets were minor. It suggests that the type of nutrition can influence mass of investigated organs. Similar observations (relative organ mass) but for gonad or pancreas mass and for iron were reported by Suliburska et al. [45, 46].

Liver steatosis was also analyzed in the tested animals. Fructose and fat diet statistically increased fat in the liver in comparison to the control group. These results were similar to observations of other authors: high-fructose diet [44, 47] and high-fat diet [48, 49]. Vanadium administration in fructose and fat diets lowered the fatty liver to the level characteristic to the control group (without additions of fructose, fat, or vanadium).

This suggests that vanadium may influence fat metabolism in the liver. Unfortunately for this moment, there are no other author works describing similar experiments. Accumulation of fat in the liver tissue may result in increased levels of liver enzymes which in consequence may be the cause of insulin resistance and glucose intolerance as a result of oversupply of fatty acids. This is a hypothesis because the share of fatty liver in the formation of insulin resistance is not fully understood. On the other hand, vanadium activity manifested in the decrease of liver steatosis is associated with their antidiabetic activity.

In the case of the spleen and the heart, we can speak only of tendencies. Fatty diet decreases the percentage of spleen weight in the whole body in both vanadium-treated and not treated rats. To better understand the interrelationships, it is necessary to determine the total body fat and muscles tissues. The increase of fat tissue usually results in the growth of body mass and in the decrease of percentage of the investigated organ mass in the whole body mass. In this study, animals were in the growth phase and the observed changes can be also associated with growth not related to fat tissue increase in the total body mass.

Vanadium treatment also influences the percentage of heart weight in all body mass where only tendencies are observed but not significant. Perhaps, a study with a bigger number of animals can show significant differences.

The reported observations for fructose and fat diet are reported by other authors. High processed food, easily assimilated sugars, excess fat (especially saturated), and little physical activity cause obesity development and are among the most important factors of civilization diseases. One of potential methods to fight this problem may be the use of vanadium compounds. Type 2 diabetes is frequently associated with insulin resistance caused by obesity. This problem is currently more and more visible in lifestyle diseases. For the moment, to achieve body mass reduction, the smallest nutrition portions and special diets are recommended. Frequently, this method in the longer term not only does not produce clear results but also produces the yo-yo effect. Investigation of substances which limit the utilization of energy from food and also normalize the glucose level, e.g., tested by us vanadium compound, will be very helpful in diabetes treatment.

## Conclusion

Vanadium methyl-bipyridine organoligand increase food and energy consumption necessary for body mass growth. Fructose and fat diet encourages faster weight gain relative to the control diet.
